# Ubiquitin specific peptidase 5 promotes ovarian cancer cell proliferation through deubiquitinating HDAC2

**DOI:** 10.18632/aging.102425

**Published:** 2019-11-13

**Authors:** Yanhua Du, Jun Lin, Rulin Zhang, Wanli Yang, Heng Quan, Lijuan Zang, Yaqin Han, Bing Li, Hong Sun, Jun Wu

**Affiliations:** 1 Department of Gynecology, Obstetrics and Gynecology Hospital of Fudan University, Shanghai 200011, P. R. China; 2 Shanghai Key Laboratory of Female Reproductive Endocrine Related Diseases, Shanghai 200011, P. R. China; 3 Pathology Center, Shanghai General Hospital, Shanghai Jiao Tong University School of Medicine, Shanghai 200080, P. R. China; 4 Department of Laboratory Medicine, Shanghai General Hospital, Shanghai Jiao Tong University, Shanghai 200080, P. R. China; 5 Department of Biochemistry and Molecular Cell Biology, Shanghai Jiao Tong University School of Medicine, Shanghai 200025, P. R. China

**Keywords:** ubiquitination, p27, epithelial ovarian cancer

## Abstract

Globally, epithelial ovarian cancer (EOC) is the most common gynecological malignancy with poor prognosis. The expression and oncogenic roles of ubiquitin specific peptidase 5 (USP5) have been reported in several cancers except EOC. In the current study, USP5 amplification was highly prevalent in patients with EOC and associated with higher mRNA expression of USP5. USP5 amplification and overexpression was positively correlated with poor prognosis of patients of ovarian serous carcinomas. Disruption of USP5 profoundly repressed cell proliferation by inducing cell cycle G0/G1 phase arrest in ovarian cancer cells. Additionally, USP5 knockdown inhibited xenograft growth in nude mice. Knockdown of USP5 decreased histone deacetylase 2 (HDAC2) expression and increased p27 (an important cell cycle inhibitor) expression *in vitro* and *in vivo*. The promoting effects of USP5 overexpression on cell proliferation and cell cycle transition, as well as the inhibitory effects of USP5 overexpression on p27 expression were mediated by HDAC2. Moreover, USP5 interacted with HDAC2, and disruption of USP5 enhanced the ubiquitination of HDAC2. HDAC2 protein was positively correlated USP5 protein, and negatively correlated with p27 protein in ovarian serous carcinomas tissues. Collectively, our data suggest the oncogenic function of USP5 and the potential regulatory mechanisms in ovarian carcinogenesis.

## INTRODUCTION

Epithelial ovarian cancer (EOC) is the most lethal gynecological malignancy [1]. Serous ovarian carcinoma accounts for about 60% of EOC and represents the most common type of EOC [2]. The currently established therapy of EOC includes surgery, platinum plus paclitaxel-based chemotherapy and radiation therapy [3]. Because of the lack of specific symptoms and effective diagnosis methods, a majority of patients are diagnosed at an advanced stage (stage III or stage IV). Although much development has been made, the prognosis for patients with advanced stage of EOC is still unfavorable. EOC represents the fifth leading cause of malignancy-related death in women worldwide [4]. Consequently, it is urgently needed to gain a better understanding of the molecular events that result in the development of EOC.

Ubiquitination is involved in a wide variety of essential biological processes in the cells, such as cell cycle progression, cell survival and DNA transcription through post-translational modification of various proteins [5]. Deubiquitinating enzymes (DUBs), a class of cysteine proteases, can regulate the disassemble of unanchored polyubiquitin and the deubiquitination of proteins. The ubiquitin specific protease family (USP), containing more than 60 members, is among the best characterized DUBs [6]. Ubiquitin specific peptidase 5 (USP5), a member of USP, was mapped to chromosome 12p13 [7, 8]. During *Drosophila* development, USP5 is reported to control ubiquitin homeostasis and the activation of Notch and receptor tyrosine kinase (RTK) signaling [9, 10]. USP5 modulates neuropathic and inflammatory pain by increasing the stability of Cav3.2 protein [11, 12]. Furthermore, it has been demonstrated that USP5 acts oncogenic roles in glioblastoma, hepatocellular carcinoma (HCC), melanoma and pancreatic cancer [13–17]. In HCC cells, USP5 knockdown inhibited cell proliferation, migration and drug resistance, while induced apoptosis and activated p14^ARF^-p53 signaling [16]. In pancreatic cancer, knockdown of USP5 up-regulated p27, attenuated G1/S phase transition, and inhibited cell proliferation [13, 14].

DNA copy-number variation (CNV) was often found to be associated with human cancers [18]. In the present study, we reported that the highly prevalent rate of USP5 gene amplification was closely associated with poor prognosis of patients with ovarian serous carcinomas. Further investigations discovered that knockdown of USP5 inhibited cell proliferation and cell cycle transition, as well as elevated p27 expression and HDAC2 ubiquitination. Our data provide new evidence for molecular function of USP5 and the potential regulatory mechanisms in ovarian carcinogenesis.

## RESULTS

### The highly prevalent rate of USP5 amplification and overall survival of patients with ovarian serous carcinomas

**Figure 1:**
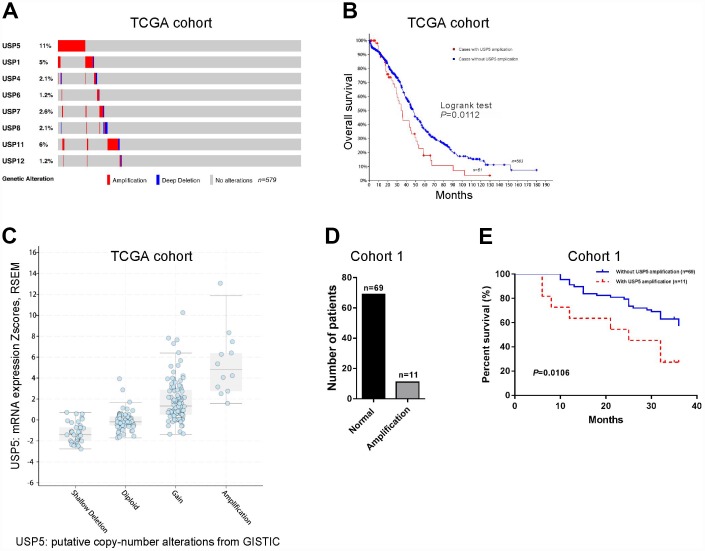
**Genomic amplification of USP5 in ovarian cancer was correlated with overall survival of patients.** (**A**) CNV analysis of USP family genes in TCGA cohort (n=579). (**B**) Kaplan-Meier survival analysis between patients with and without USP15 amplification using TCGA cohort (n=564). (**C**) USP15 mRNA levels were higher in samples with USP5 amplification than in those without USP5 amplification. (**D**) USP5 copy number alteration in patients of cohort 1 by real-time PCR analysis (n=80). The cut-off for amplification was set at 4 copies per tumor cell. (**E**) Kaplan-Meier survival analysis between patients with and without USP15 amplification using cohort 1 (n=80).

CNV analysis performed on TCGA ovarian serous carcinomas dataset revealed that 8 members of USP displayed copy-number amplification in patients with ovarian serous carcinomas (n=579), and USP5 had the highest amplification rate (Figure 1A). Further Kaplan-Meier survival analysis showed that patients with USP5 amplification had shorter survival time than those without USP5 amplification (P<0.05). Therefore, we focused on USP5 in this study. The effects of USP5 CNV on mRNA expression were then evaluated by GISTIC analysis, and the results showed that USP5 amplification was associated with higher mRNA expression of USP5 in ovarian serous carcinomas patients (Figure 1C). Further, CNV detection was performed on cohort 1 patients from our own hospital (n=80). Real-time PCR showed that USP5 amplification was 13.8% of patients when a cut-off was set at 4 copies per tumor cell (Figure 1D). Survival analysis on cohort 1 also confirmed the prognostic value of USP5 amplification in ovarian serous carcinomas (Figure 1E, P<0.05). The median overall survival time for patients with USP5 amplification was 25 months, while the median overall survival time for patients without amplification was undefined due to the short duration time of follow-up.

### USP5 was up-regulated in ovarian serous carcinomas tissues and high USP5 expression predicted poor prognosis

**Figure 2:**
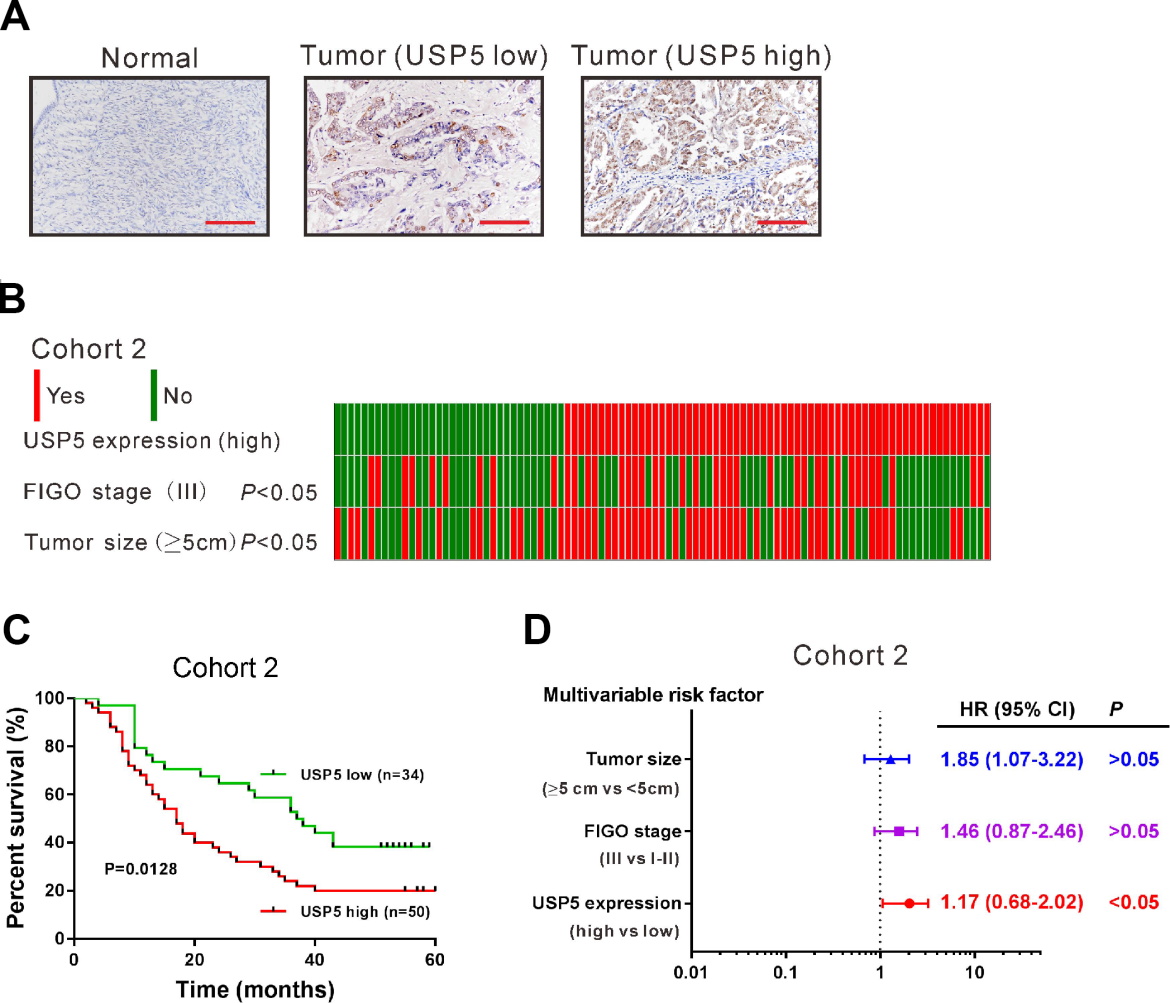
**USP5 was up-regulated in ovarian cancer tissues and associated with overall survival.** (**A**) Immunohistochemistry staining of USP5 in ovarian cancer and noncancerous tissues from cohort 2. Scale bar: 100 μm. The patients were divided into two groups USP5 low expression and USP5 high expression with a cut-off of 25% of positively-stained cancer cells. (**B**) Analysis of correlation between USP5 expression, FIGO stage and tumor size in cohort 2. (**C**) Kaplan-Meier survival analysis of ovarian cancer patients with high expression of USP5 and low expression of USP5 (log-rank analysis). (**D**) Multivariate regression analysis in cohort 2.

We then detected USP5 protein expression by immunohistochemical staining in 84 ovarian serous carcinomas tissues and 12 noncancerous ovary tissues (cohort 2). The upregulation of USP5 protein was also observed in ovarian serous carcinomas tissues. These 84 EOC cases were then divided into USP5 low expression group (n=34) and high expression group (n=50) (Figure 2A) based on the positive staining ratio of cancer cells. Chi-square test indicated that there is a close correlation between USP5 expression and tumor size and FIGO stage (Figure 2B, *P*<0.05). Kaplan-Meier survival analysis revealed that high expression of USP5 was closely associated with poor overall survival of patients with ovarian serous carcinomas (Figure 2C). The median overall survival time for patients with USP5 low expression and high expression was 37 months and 17 months, respectively. Multivariate Cox regression analysis revealed that USP5 expression was an independent prognostic marker for ovarian serous carcinomas (Figure 2D, P<0.05). These data suggested that USP5 was up-regulated in ovarian serous carcinomas specimens and significantly correlated with poor prognosis.

### Down-regulation of USP5 inhibited cell proliferation of ovarian cancer cells

**Figure 3:**
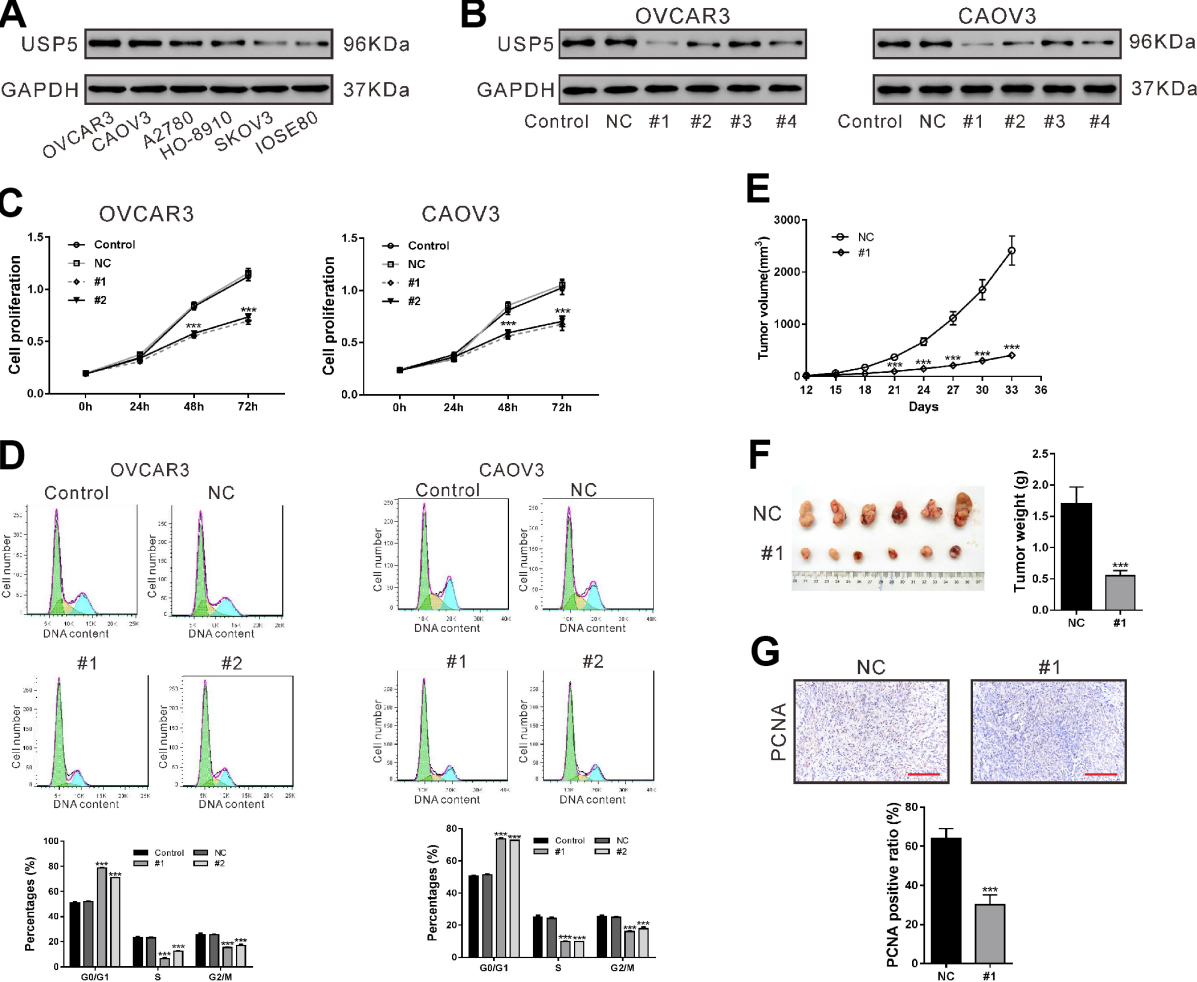
**Down-regulation of USP5 inhibited cell proliferation and cell cycle progression of ovarian cancer cells.** (**A**) Western blotting analysis of USP5 in 5 ovarian cancer cell lines and a normal human ovarian cell line IOSE80. (**B**) OVCAR3 and CAOV3 cells were transiently infected with USP5 shRNAs (#1, #2, #3 and #4), control shRNA (NC) or untreated (Control). Western blotting analysis was performed to check the knockdown efficiency of USP5 shRNAs. (**C**) Proliferation of OVCAR3 and CAOV3 cells expressing NC or USP5 shRNAs (#1, #2) was detected at the indicated time points by CCK-8 assay. (**D**) Cell cycle distribution of OVCAR3 and CAOV3 was assessed at 48 h after virus infection by PI staining and flow cytometry analysis. Representative graphs and statistical analysis of percentages at different cell cycle stages are shown. (**E**–**G**) Nude mice were transplanted with OVCAR3 cells expressing control (NC) or USP5 shRNA (#1). (**E**) Tumor volume was assessed at the indicated time points in both group (n=6 per group). (**F**) On 33 days after transplantation, tumors were resected and weighed. (**G**) Immunohistochemistry staining of PCNA in xenografts. Scale bar: 100 μm. Representative images and statistical analysis of percentages are shown. ***P<0.001.

We examined the USP5 protein levels in five ovarian cancer cell lines and a normal human ovarian cell line IOSE80 (Figure 3A). Two ovarian cancer cell lines, OVCAR3 and CAOV3, showed the highest protein expression of USP5, while SKOV3 had similar USP5 expression as IOSE80 cells. To explore the functional relevance of the upregulation of USP5 in ovarian cancer cells, we used lentivirus-mediated shRNAs to knock down USP5 expression in OVCAR3 and CAOV3 cells, which expressed relatively high levels of USP5. Both cells were transduced with USP5 shRNAs (#1, #2, #3 and #4) or control shRNA (NC) and cells without any treatment were served as Control group. After 48 h, western blotting was carried out to assess USP5 protein level. As shown in Figure 3B, NC had little effect on USP5 expression compared to the Control group, and USP5 shRNAs (#1, #2 and #4) reduced USP5 protein expression in both cell lines.

Following USP5 shRNAs (#1 and #2) transduction, the proliferation of both cancer cell lines was determined using CCK-8 assays, which showed that down-regulation of USP5 caused profoundly reduced proliferation of both ovarian cancer cell lines (Figure 3C, P<0.001).

### Down-regulation of USP5 induced G0/G1 arrest of ovarian cancer cells

To unravel the mechanisms underlying the inhibition of cell proliferation, we analyzed cell cycle distribution of ovarian cells after USP5 knockdown using PI staining and flow cytometry analysis. As shown in Figure 3D, 78.53 ± 0.36% and 70.96 ± 0.31% of cells were at G0/G1 phase in USP5 shRNA#1 and #2 infected OVCAR3 cells, respectively, which were profoundly higher than those of Control cells (51.06 ± 0.53%, P<0.001) and NC infected cells (52.02 ± 0.38%, P<0.001). Meanwhile, there were less cells at S phase and G2/M phase after USP5 shRNA#1 and #2 infection, compared with control cells and NC infected cells (P<0.001). Similar results were observed in CAOV3 cells. These results indicated that USP5 knockdown induced cell cycle arrested at G0/G1 phase in ovarian cancer cells.

### Down-regulation of USP5 inhibited xenograft growth in nude mice

Next, we investigated the effect of USP5 on the tumorigenic potential of OVCAR3 cells. Knockdown of USP5 (#1) led to significant decrease in tumor growth rate of OVCAR3-derived xenografts at the interval between Day 21 and Day 33 after cell transplantation (Figure 3E, P<0.001). On Day 33, the xenografts were collected and weighed, which showed that the tumor weight was also reduced by USP5 knockdown (Figure 3F, P<0.001). Immunochemistry staining results showed that the ratio of PCNA positive cells was significantly lower with USP5 knockdown (Figure 3G, P<0.001). These data indicated that USP5 knock-down ovarian cancer cells had lower proliferation rate *in vivo*.

### USP5 knockdown induced p27 expression via repressing HDAC2 expression

**Figure 4:**
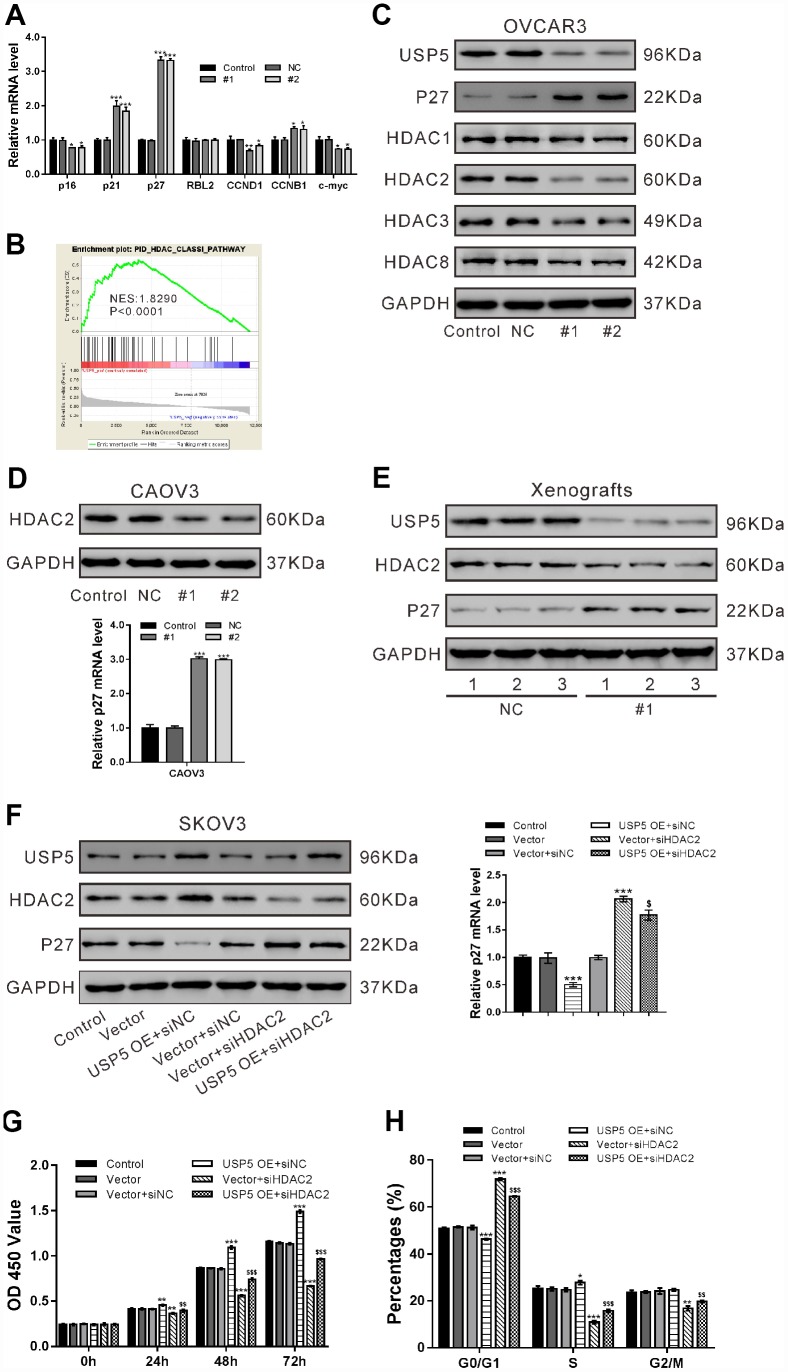
**USP5 knockdown induced p27 expression via repressing HDAC2 expression.** (**A**) OVCAR3 cells were transiently infected with USP5 shRNAs (#1 and #2), control shRNA (NC) or untreated (Control). Real-time PCR analysis of p16, p21, p27, RBL2, CCND1 and c-Myc. (**B**) GSEA analysis in ovarian cancer patients with higher USP5 expression versus lower USP5 expression (TCGA dataset). NES, normalized enrichment score. (**C**) Western blot analysis of HDAC Class I members and p27. (**D**) Western blotting and real-time PCR analyses were performed to check the expression of HDAC2 and p27 in CAOV3 cells expressing NC or USP5 shRNAs (#1, #2). (**E**) The protein expression of USP5, HDAC2 and p27 in xenografts formed from OVCAR3 cells expressing control (NC) or USP5 shRNA (#1). Three samples were randomly chosen from each group. (**F**-**H**) SKOV3 cells were infected with USP5 overexpressing virus (USP5 OE) or control virus (Vector), and treated with HDAC2 siRNA (siHDAC2) or control siRNA (siNC) as indicated. The protein expression of USP5, HDAC2 and p27 was detected, and mRNA expression of p27 was evaluated at 48 h after treatment (**F**). Proliferation (**G**) and cell cycle distribution (**H**) was evaluated by CCK-8 and PI/flow cytometry analysis, respectively. *P<0.05,**P<0.01, ***P<0.001 vs. Vector+siNC; $$P<0.01, $$$ P<0.001 vs. USP5 OE+siNC (ANOVA test followed by a Tukey post hoc test).

To figure out the possible mechanisms underlying the inhibitory effects of USP5 knockdown on cell proliferation, we analyzed the mRNA expression of proliferation-related genes including p16, p21, p27, RBL2, CCND1, CCNB1 and c-myc in OVCAR3 cells. Real-time PCR analysis suggested that p21 and p27, important cell cycle inhibitors [19], increased the most in the cells with USP5 knockdown (Figure 4A).

By analyzing TCGA ovarian serous carcinomas expression dataset, we found that HDAC Class I pathway was significantly correlated with USP5 expression (Figure 4B). We then analyzed the protein levels of HDAC Class I proteins (HDAC1, −2, −3, and −8) in OVCAR3 cells, and found that USP5 knockdown decreased the expression of HDAC2 at translational level (Figure 4C).

The inhibitory effects of USP5 knockdown on HDAC2 expression and its induced effects on p27 expression was also observed in CAOV3 cells (Figure 4D) and xenografts (Figure 4E). Previous studies have documented the regulation role of HDAC2 on p27 [20–22]. To determine whether HDAC2 mediated the regulation of USP5 on p27, we treated SKOV3 cells with USP5 overexpressing virus (USP5 OE) or HDAC2 siRNA (siHDAC2) and evaluated p27 expression levels. As shown in Figure 4F, USP5 overexpression led to a profound decrease in p27 levels and an obvious induction in HDAC2 levels, while HDAC2 knockdown had reverse effect. The cells treated with USP5 OE and siHDAC2 showed the similar expression levels of HDAC2 and p27 as those treated with Vector and control siRNA (siNC), indicating that USP5 down-regulated p27 expression via regulating HDAC2.

Further, CCK-8 (Figure 4G) and flow cytometry analyses (Figure 4H) showed that HDAC2 knockdown partially abrogated cell proliferation promoted by USP5 overexpression. These data indicated that USP5 promoted ovarian cancer cell proliferation and cell cycle progression through regulating HDAC2.

### USP5 regulated HDAC2 ubiquitination

**Figure 5:**
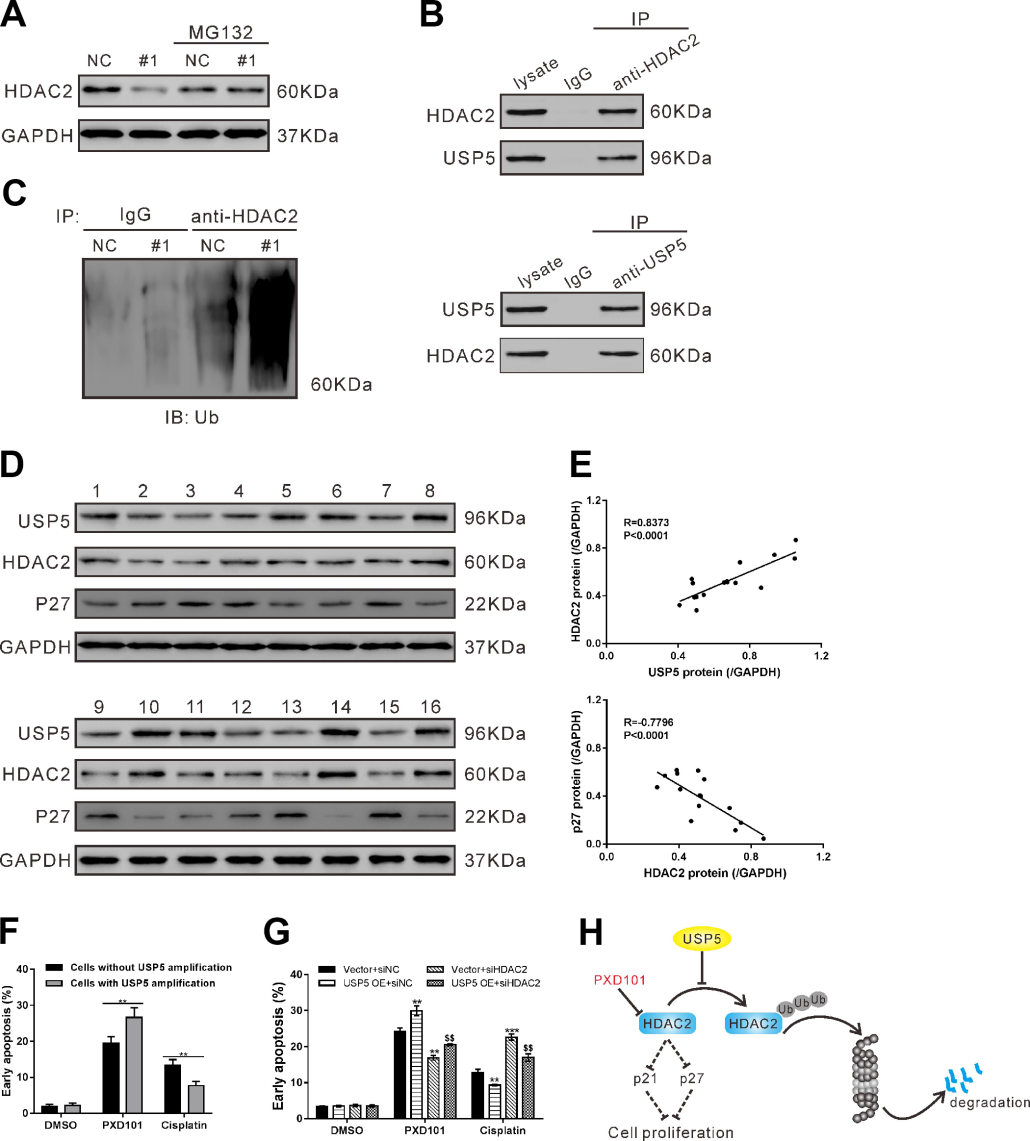
**USP5 regulated HDAC2 ubiquitination.** (**A**) OVCAR3 cells were infected with USP5 shRNA (#1) or control shRNA (NC) in the presence and absence of 10 μM MG132 for 48 h. The protein expression of HDAC2 was assessed. (**B**) Cell lysates from OVCAR3 cells were immunoprecipitated (IP) with anti-HDAC2 or anti-USP5 or IgG, and then western blotting analysis was performed with anti-HDAC2 or anti-USP5. (**C**) Cell lysates from OVCAR3 cells infected with USP5 shRNA (#1) or control shRNA (NC) were IP with anti-HDAC2 or IgG, and western blotted with anti-ubiquitin. (**D**) Western blotting analysis of USP5, HDAC2 and p27 expression in ovarian cancer tissues from cohort 2 (n=16). (**E**) Pearson correlation scatter plots showed a positive correlation between USP5 and HDAC2, and a negative correlation between p27 and HDAC2. (**F**) Early apoptosis induced by PXD101 (1 μM) or cisplatin (5 μg/ml) in primary ovarian cancer cells with or without USP5 amplification. (**G**) SKOV3 cells were infected with USP5 overexpressing virus (USP5 OE) or control virus (Vector), and treated with HDAC2 siRNA (siHDAC2) or control siRNA (siNC) as indicated for 24 h. Early apoptosis induced by PXD101 (1 μM) or cisplatin (5 μg/ml) was detected at 48 h post treatment. **P<0.01, ***P<0.001 vs. Vector+siNC; $$P<0.01 vs. USP5 OE+siNC (ANOVA test followed by a Tukey post hoc test). (**H**) A working model that USP5 plays a key role in cell proliferation by inhibiting HDAC2 ubiquitination.

USP5 is a member of deubiquitinases [7]. In OVCAR3 cells, MG132 treatment suppressed the inhibitory effects of USP5 knockdown on HDAC2 protein (Figure 5A), which suggested that a proteasome-dependent pathway was involved in the regulation of USP5 on HDAC2 protein. Immunoprecipiation experiments with antibody against USP5 or HDAC2 showed the interaction between USP5 and HDAC2 in OVCAR3 cells (Figure 5B), and that USP5 knockdown induced the ubiquitination of HDAC2 (Figure 5C). These data indicated that USP5 knockdown caused a down-regulation of HDAC2 through a post-translational modification.

The protein expression of USP5, HDAC2 and p27 was assessed in 16 ovarian serous carcinomas tissues of cohort 2 (Figure 5D). Pearson correlation analysis revealed that HDAC2 protein expression was positively correlated with USP5 protein expression, and negatively correlated with p27 protein expression in ovarian serous carcinomas tissues (Figure 5E, P<0.0001).

### USP5 amplification increased the proapoptotic effect of HDAC inhibitor PXD101 in primary ovarian cancer cells

HDAC inhibitors have been proved to be effective in inhibiting cancer cell proliferation and inducing cancer cell death, and have been developed as potential anti-cancer therapeutics [23]. Considering the association between USP5 and HDAC2, we hypothesized that USP5 amplification may affect the sensitivity of ovarian cancer cells to HDAC inhibitor. To test our hypotheses, primary ovarian cancer cells were isolated from 8 patients. Of these cells, 3 cells showed USP5 amplification. The primary ovarian cancer cells were then exposed to PXD101, cisplatin or DMSO for 48 h. As shown in Figure 5F, cells with USP5 amplification exhibited a greater rate of cell apoptosis than in those without USP5 amplification when PXD101 treatment was applied, while cells with USP5 amplification were more resistant to cisplatin-induced apoptosis.

To further study the association of USP5 and HDAC2 in chemosensitivty, SKOV3 cells with USP5 overexpressing virus (USP5 OE) or HDAC2 siRNA (siHDAC2) were also exposed to PXD101, cisplatin or DMSO. As shown in Figure 5G, PXD101 treatment induced a greater rate of apoptosis in cells overexpressing USP5 than in cells expressing control Vector, while cisplatin exhibited reversed effects. Moreover, HDAC2 knockdown rescued the effects of USP5 overexpression.

## DISCUSSION

In the present study, CNV analysis on USP family members revealed that USP5 amplification was common in ovarian serous carcinomas and that patients with USP5 amplification had a poor prognosis compared to patients without USP5 amplification. To our knowledge, this is the first report concerning the relation between USP5 CNV and carcinogenesis. USP5 expression is up-regulated in hepatocellular carcinoma [16] and pancreatic cancer [13, 14]. Consistent with these findings, our clinical pathological analysis showed that USP5 was relatively elevated in cancer tissues compared with corresponding noncancerous tissues. USP5 expression showed a positive correlation with tumor size, tumor grade and poor prognosis of ovarian serous carcinomas patients. Our experiments further confirmed that USP5 knockdown could suppress ovarian cell proliferation both *in vitro* and *in vivo*. USP5 knockdown significantly induced G0/G1 arrest in ovarian cancer cells. These findings together with previous studies in glioblastoma, HCC, melanoma and pancreatic cancer [13–17] demonstrated the oncogenic roles of USP5.

We also investigated the molecular mechanism for the oncogenic roles of USP5 in EOC. Previous studies indicated that USP5 acts as a deubiquitinase for p53 [17, 24] and Cav3.2 protein [11, 12]. We then tried to identify the target proteins of USP5 in ovarian carcinogenesis. By Gene set enrichment analysis (GSEA) of TCGA ovarian serous carcinomas dataset, HDAC Class I pathway was found to be significantly correlated with USP5 expression. Western blotting analysis showed that the most affected Class I HDAC member in ovarian cancer cells with USP5 knockdown was HDAC2. Upregulation of HDAC2 is frequently observed in human tumor tissues [25–29], and knockdown of HDAC2 suppresses tumor cell proliferation and tumor progression [29–31]. It has been stated that ubiquitin-proteasome system is important regulator for HDAC2 stability. The E2 ubiquitin conjugase Ubc8 and the E3 ubiquitin ligase RLIM participate in the proteasomal degradation of HDAC2 induced by the Class I HDAC inhibitor, valproic acid (VPA) [32]. MULE (Mcl-1 ubiquitin ligase E3) directly targets HDAC2 for ubiquitination and degradation [33]. Recently, two members of USPs, USP17 [34] and USP4 [35], have been reported to deubiquitinate HDAC2. In our study, USP5 knockdown inhibited HDAC2 expression *in vitro* and *in vivo*. HDAC2 interacted with USP5, and USP5 knockdown induced the ubiquitination of HDAC2. HDAC2 knockdown partially abrogated USP5-promoted ovarian cancer cell proliferation. In ovarian serous carcinomas tissues, a positive correlation was observed between the protein expression of USP5 and HDAC2. Additionally, knockdown of USP5 or HDAC2 up-regulated p27, which was consistent with the previous reports [13, 14, 20–22]. p21, another cell cycle inhibitor [19], was negatively regulated by HDAC2 [3]. Although the increase ratio of p21 mRNA in cells with USP5 knockdown (Figure 4A) was less than that of p27, USP5/HDAC2 had the same regulatory effects on p21 expression in the present study (Supplementary Figure 1). Thus, we speculate that USP5 may be a deubiquitinase of HDAC2, which negatively regulated the expression of p27 and p21 and led to the accelerated cell cycle transition and cell proliferation in ovarian cancer cells (Figure 5H). Further, HDAC inhibitors are more and more frequently applied for cancer treatment [37]. USP5 overexpression increased the sensitivity of ovarian cancer cells to HDAC inhibitor PXD101, which was reversed by HDAC2 knockdown (Figure 5G). We found that ovarian cancer cells with USP5 amplification was more sensitive to PXD101-induced apoptosis than those without USP5 amplification (Figure 5F). These data suggested that the detection of USP5 amplification should be considered before the anti-cancer therapy by targeting HDAC was applied to ovarian serous carcinomas patients, although further animal experiments and clinical trial are needed.

In summary, this is the first study to investigate a molecular link connecting USP5, HDAC2, and p27 in ovarian serous carcinomas. The data of this study indicate that USP5 may up-regulate the protein levels of HDAC2 through suppressing its ubiquitination, which leads to the down-regulation of p27 and the progression of EOC. Importantly, targeting USP5 might bring novel therapeutic options for ovarian serous carcinomas.

## MATERIALS AND METHODS

### Human specimen collection

The study was approved by the local, independent ethics committees at Shanghai general hospital and Obstetrics and Gynecology Hospital of Fudan University. Two cohorts of ovarian cancer patients treated at Shanghai General Hospital (cohort 1) and Obstetrics and Gynecology Hospital of Fudan University (cohort 2) were enrolled in this study. All the patients underwent surgery and the patients at stage II/III received 6–8 cycles of a paclitaxel/carboplatin combination therapy after surgery. Written informed consent was obtained from all participants. Cohort 1 contained 80 patients treated between January 2014 and December 2015 and follow-up lasted for three years, and ovarian serous carcinomas specimens were collected from these patients and subjected to CNV detection. Cohort 2 included 84 patients between January 2010 and December 2012 with clinicopathologic features (Table 1) and follow-up lasted for five years. Formalin-fixed paraffin-embedded sections were prepared from all the samples and subjected to immunohistochemistry staining. Among the 84 ovarian serous carcinomas cases, ovarian cancer tissues from 16 cases were immediately frozen in liquid nitrogen, kept at −80 °C and available for western blot analysis.

**Table 1:** Clinicopathologic features in cohort 2.

**Clinicopathologic features**	**n**	**%**
Age (years)		
<60	55	65.5
≥60	29	34.5
Histologic type		
Serous	48	57.1
Endometrioid	13	15.5
Mucinous	11	13.1
Clear cell	8	9.5
Mixed	4	4.8
FIGO stage		
I-II	47	56.0
III	37	44.0
Tumor size		
<5cm	32	38.1
≥5cm	52	61.9

### Bioinformatics analysis

The Cancer Genome Atlas (TCGA) ovarian serous carcinomas dataset was obtained at TCGA website (https://tcga-data.nci.nih.gov/tcga). The DNA copy number and the effects of USP5 CNV on mRNA expression were evaluated by GISTIC analysis and one-side Jonckheere-Terpstra test, respectively. Overall survival analysis was performed using Kaplan–Meier method and log-rank test.

Gene set enrichment analysis (GSEA) was performed with TCGA ovarian serous carcinomas expression dataset as previously described by using GSEA version 2.0 from the Broad Institute (http://www.broad.mit.edu/gsea) [38]. Gene set permutations were performed 1000 times, and the pathway set list is sorted by the normal P value and the Normalized Enrichment Score (NES).

### CNV detection

CNV was quantitatively analyzed as previously described [39, 40]. Genomic DNA was isolated form 80 ovarian cancer specimens (cohort 1) by using TGuide S32 Magnetic Tissue DNA Kit (TIANGEN, Shanghai, China) as the manufacturer suggested. DNA concentration was measured using NanoDrop 1000 (Thermo Scientific, Rockford, IL, USA). The copy number of USP5 gene was detected with QX200 Droplet Digital PCR System (Bio-Rad, Richmond, CA, USA) according to the manufacturer’s instructions. The target probe was as follows: 5′ -TCCCCTGCCCTCATT GGTAAGGGGT- 3′.

### Cell culture

All the cell lines were obtained from the Institute of Biochemistry and Cell Biology, Chinese Academy of Sciences (Shanghai, China) and maintained in a humidified atmosphere containing 5% CO_2_ at 37 °C. OVCAR3, A2780 and HO-8910 cells were grown in RPMI 1640 (Hyclone, Logan, UT, USA), while CAOV3, SKOV3, IOSE80 and 293T cells were cultured in Dulbecco’s modified Eagle’s medium (DMEM; Hyclone). All culture media were supplemented with 10% fetal bovine serum (FBS; Invitrogen, Carlsbad, CA, USA), 100 mg/ml penicillin G and 50 μg/ml streptomycin.

### Immunohistochemistry staining

Tissue sections were routinely deparaffinized and rehydrated. Antigen-retrieval was performed by heating the sections in 0.01M citrate buffer (pH 6.0) for 15 min. After blocking endogenous peroxidase activity and non-specific antigens, the sections were incubated with rabbit anti-USP5 (Abcam, Cambridge, MA, USA) overnight at 4 °C, and then with goat anti-rabbit secondary antibody at room temperature for 1 h. The sections were developed with 3,3′-diaminobenzidine solution, and the nuclei were counterstained with hematoxylin. The sections were scored separately by two pathologists. The patients were divided into two groups: USP5 low expression, less than 25% of cancer cells showed positive stain; USP5 high expression, more than 25% of cancer cells showed positive stain.

### Western blotting

Frozen tissues (0.2 g per sample) were grinded into powder using mortar and pestle in liquid nitrogen. Frozen tissue powder and cultured cells were lysis with radioimmunoprecipitation assay buffer (RIPA) supplemented with proteinase inhibitor cocktail (Sigma, St. Louis, MO, USA). Protein extracts were quantified with BCA assay kit (Thermo Fisher Scientific, Rockford, IL, USA), and loaded onto 10% or 12% sodium dodecyl sulfate–polyacrylamide gel electrophoresis (30 μg of protein per sample). After transferring to nitrocellulose membranes and blocking with 5% skim milk, samples were probed with primary antibodies overnight at 4 °C followed by horseradish peroxidase-linked secondary antibodies (Beyotime, Shanghai, China) for 1 h at 37°C. Signal was visualized using enhanced chemiluminescence (ECL, Millipore, Bredford, USA). GAPDH was detected as loading control. The information of primary antibodies is listed in Supplementary Table 1.

### Real-time PCR

Total RNA was isolated with Trizol reagent (Invitrogen), and real-time PCR was performed with the SYBR Green Kit (Thermo Fisher Scientific) and ABI 7300 System (Applied Biosystems, Foster City, CA, USA). The information of primers is listed in Supplementary Table 2.

### Manipulation of USP5 expression in ovarian cancer cell lines

To knock down USP5 expression, short hairpin RNAs (shRNAs) targeting human USP5 mRNA (1#, GCTCTC AAGGGAATCTTTA; 2#, GGATATATGTACTGCCC AA; 3#, CCTACTATACTCCCAACTT; 4#, CCACTT GCTCAGTCGTCAA) and a specific scramble shRNA (NC) were cloned into pLKO.1 lentiviral vector (Addgene, Cambridge, MA, USA).

To ectopic express USP5, human USP5 cDNA was amplified with the following primers: forward, 5′ -CGGAATTC ATGGCGGAGCTGAGTGAGGAG-3′ and reverse, 5′-CGGGATCCTAGCTGGCCACTCTCT GG-3′, and cloned into pLVX-puro lentiviral vector (Clontech, Palo Alto, CA, USA).

For lentivirus production, 293T cells were transfected with lentiviral vector and packaging vectors using Lipofectamine 2000 (Invitrogen, Carlsbad, CA, USA). And 48–72 h later, viruses were collected from culture media, filtered and infected indicated ovarian cancer cell lines.

### Silencing of HDAC2 (histone deacetylase 2) by small interfering RNA (siRNA)

HDAC2 siRNA (siHDAC2: 5′- GGUCAAUAAGACC AGAUAAUU -3′) and a non-specific scramble siRNA (siNC: 5′- UUGUACUACACAAAAGUACUG-3′) were synthesized by Genepharma (Shanghai, China) and transiently transfected into SKOV3 cells with Lipofectamine 2000 (Invitrogen) as per the manufacture’s instruction.

### Cell count Kit-8 (CCK-8) assay

CCK-8 assay was done to analyze ovarian cancer cell proliferation. Cells were plated onto 96-well plates (3000 cells per well) and treated as indicated. After 0, 24, 48 and 72 h, CCK-8 (SAB biotech. College Park, MD, USA) solution was added to each well and incubated for 1 h. The optical density at 450 nm (OD 450) of each well was measured using a multilabel plate reader. Experiments were repeated three times.

### Cell cycle distribution analysis

Cells were treated as indicated. About 48 h later, the cells were trypsinized, washed with ice-cold PBS and fixed in 70% ethanol overnight at −20°C. Subsequently, the fixed cells were washed with PBS and stained with propidium iodide (PI) solution (50 μg/mL PI and 100 μg/mL RNase A in PBS) in the dark for 30 min at 37 °C. DNA content was measured on a FACScan flow cytometer (BD Biosciences, San Jose, CA, USA) with FlowJo software (version 7.6.1, Tree Star, Ashland, OR, USA). Experiments were repeated three times.

### Cell apoptosis analysis

To test whether USP5 amplification affects the proapoptosis activity of PXD101, primary ovarian cancer cells were isolated from 8 patients who were admitted to Shanghai General Hospital as previously described [41] after written informed consent was obtained. Genomic DNA was isolated from these cells and CNV was detected as described above. These cells were seeded into 6-well plates, cultured overnight and exposed to PXD101 (1 μM; Selleck Chemicals, Houston, TX, USA), cisplatin (5 μg/ml; Aladdin, Shanghai, China) or vehicle (DMSO). After 48 h, cell apoptosis was assessed by Annexin V-FITC detection Kit (Beyotime, Shanghai, China) according to the manufacturer’s protocol.

### *In vivo* tumorigenesis in nude mice

Animal experiments were approved by the Animal Care Committee at Shanghai Jiaotong University. Four-week-old male BALB/c nude mice (SLAC laboratory animal Center, Shanghai, China) were housed in specific-pathogen-free condition and randomly divided into two groups (n=6 per group). Logarithmically growing OVCAR3 cells expressing USP5 shRNA (#1) or control shRNA (NC) were collected and adjusted to a density of 5 × 10^7^/mL in PBS. Every nude mouse was subcutaneously injected with 0.1 mL cell suspension on Day 1. Tumor volume was measured every three days since the tumors were visible (Day 12). On Day 33, the mice were sacrificed and the xenografts were weighed. Parts of the xenografts were immediately frozen and kept at −80 °C for further western blot analysis, and remaining tissues were processed for formalin-fixed paraffin-embedded-sectioned and immunohistochemistry staining with anti-PCNA (Abcam).

### Coimmunoprecipitation assays

Cell lysates were incubated with anti-USP5 (Abcam), anti-HDAC2 (Abcam) or control IgG (Santa Cruz Biotech., Santa Cruz, CA, USA) for 1 h at 4°C with rotation, and then with protein A/G-agarose (Santa Cruz Biotech.) for 2 h at 4°C with rotation. Precipitates were washed three times in lysis buffer to remove non-specific binding, denatured in sample buffer and detected by western blot analysis with indicated primary antibodies.

### Statistical analysis

GraphPad Prism software (version 6.0, GraphPad Software, La Jolla, CA, USA) was applied for statistical analysis. Fisher’s exact test was used to assess the correlation between USP5 protein expression and clinicopathological features. ANOVA test was used for comparisons among groups. P<0.05 is considered statistically significant.

## Supplementary Material

Supplementary Figure 1

Supplementary Tables
