# Supplementary material for: Ubiquitin specific peptidase 5 promotes ovarian cancer cell proliferation through deubiquitinating HDAC2
Source: Aging (Albany NY). 2019 Nov 13;11(21):9778–93. doi: 10.18632/aging.102425 (PMC6874447; doi:10.18632/aging.102425)
Supplement: Supplementary Figure 1 [file aging-11-102425-s002.pdf]

SUPPLEMENTARY FIGURE

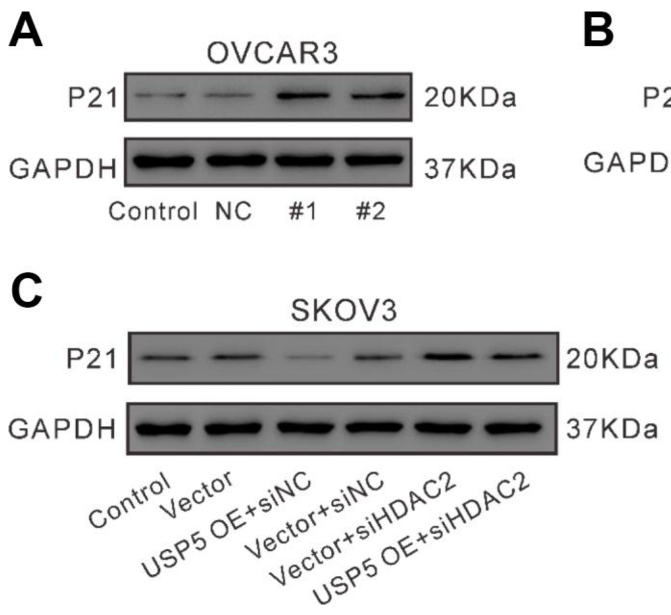

Supplementary Figure 1. Western blots for p21. (**A**) OVCAR3 cells were transiently infected with USP5 shRNAs (#1 and #2), control shRNA (NC) or untreated (Control). The protein expression of p21 was detected. (**B**) The protein expression of p21 in xenografts formed from OVCAR3 cells expressing control (NC) or USP5 shRNA (#1). Three samples were randomly chosen from each group. (**C**) SKOV3 cells were infected with USP5 overexpressing virus (USP5 OE) or control virus (Vector), and treated with HDAC2 siRNA (siHDAC2) or control siRNA (siNC) as indicated. The protein expression of p21 was detected.
