# Supplementary material for: Ubiquitin specific peptidase 5 promotes ovarian cancer cell proliferation through deubiquitinating HDAC2
Source: Aging (Albany NY). 2019 Nov 13;11(21):9778–93. doi: 10.18632/aging.102425 (PMC6874447; doi:10.18632/aging.102425)
Supplement: Supplementary Tables [file aging-11-102425-s001.pdf]

SUPPLEMENTARY TABLES

Supplementary Table 1. Antibody information.

Primary antibody	Company	Catalog No.
USP5	Abcam (Cambridge, MA, USA)	Ab235772
HDAC1		Ab53091
HDAC2		Ab32117
HDAC3		Ab32369
HDAC8		Ab187139
GAPDH	Cell Signaling Technology (Danvers, MA, USA)	#5174
P21		#2947
P27		#2552

Supplementary Table 2. Primer sequence for real-time PCR.

Primer	Primer sequence	Size (bp)
p16	F: 5′-GGTGCCACATTCGCTAAG -3′<br>R: 5′-ACCCTGTCCCTCAAATCC -3′	116
p21	F: 5′-TAGCAGCGGAACAAGGAG -3′<br>R: 5′-AAACGGGAACCAGGACAC -3′	249
p27	F: 5′- CAGCAGCTACAGCACTAAG -3′<br>R: 5′- AGAGTGGAGAGGTGAAGAG -3′	157
RBL2	F: 5′- GGCGGCTATTTGTTGAGAATG -3′<br>R: 5′- TGACTTGGACAGGGAAGAATG -3′	255
CCND1	F: 5′- TTCGTGGCCTCTAAGATG -3′<br>R: 5′- GTGTTTGCGGATGATCTG -3′	222
CCNB1	F: 5′- CTCCGGTGTTCTGCTTCTC -3′<br>R: 5′- GCTGTTCTTGGCCTCAGTC -3′	183
c-Myc	F: 5′- CCTTCTTTCCTCCACTCTC -3′<br>R: 5′- CAAACCCTCTCCCTTTCTC -3′	231
GADPH	F: 5′- AATCCCATCACCATCTTC -3′<br>R: 5′- AGGCTGTTGTCATACTTC -3′	218
